# The relationship between social exclusion and meaning in life among college students: a moderated mediation model

**DOI:** 10.3389/fpsyg.2026.1879039

**Published:** 2026-07-22

**Authors:** Fengjiang Yue

**Affiliations:** School of Law (Humanities and Social Sciences), Taiyuan University of Science and Technology, Taiyuan, China

**Keywords:** college student, loneliness, meaning in life, nostalgia, social exclusion

## Abstract

**Objective:**

To explore the relationship between social exclusion and meaning in life among college students, as well as the roles of loneliness and nostalgia in this relationship.

**Methods:**

From August to October 2025, a sample of 1714 college students from a university in Taiyuan City was assessed using the Social Exclusion Questionnaire for College Students, the Loneliness Scale, the Indigenous Nostalgia Scale, and the Meaning in Life Scale.

**Results:**

(1) Social exclusion significantly and negatively predicted college students’ meaning in life. (2) Loneliness partially mediated the relationship between social exclusion and meaning in life. (3) Nostalgia significantly moderated the direct path, the first half of the mediation path (social exclusion → loneliness), and the second half of the mediation path (loneliness → meaning in life) in the mediation model.

**Conclusion:**

Social exclusion can negatively predict college students’ meaning in life both directly and indirectly through loneliness. Nostalgia moderates the direct effect of social exclusion on meaning in life as well as the first and second halves of the mediating process.

## 1 Introduction

Social exclusion refers to the negative interpersonal experience in which an individual is rejected, ignored, or ostracized by other individuals or groups (Williams, 2007). In the unique and critical developmental stage of college, peer acceptance plays a vital role in students’ psychological adjustment and personality formation. However, college students are precisely a high-risk group for social exclusion, as they have left their original family and friendship support systems and need to rebuild social relationships in an entirely new environment. Research has shown that social exclusion exerts a broad range of detrimental effects on college students’ mental health and development. It not only triggers intense negative emotions, such as depression (Lui et al., 2020), but also leads to decreased self-esteem (Lei et al., 2023). In the long term, persistent social exclusion significantly elevates college students’ levels of anxiety and negative affect (Jia et al., 2019), and may impair academic performance through damage to executive functions (Yu et al., 2025). Given the multiple adverse impacts of social exclusion on college students’ development, a thorough investigation of its underlying mechanisms is of critical theoretical and practical significance for safeguarding college students’ mental health and designing effective psychological interventions.

Meaning in life refers to the extent to which individuals perceive their lives as meaningful, including their sense of life purpose and primary goals (Wang et al., 2026). Research indicates that social relationships serve as a crucial foundation for meaning in life (Chen and Li, 2015; Ma et al., 2023), and social support can enhance individuals’ existential experience by increasing feelings of happiness and satisfaction (Jadidi and Ameri, 2022). However, social exclusion poses a serious threat to this process, as it can trigger cognitive deconstruction in individuals, leading them to fall into a state of meaninglessness (Twenge et al., 2003). Such exclusion experiences impair individuals’ sense of control over their lives and their self-worth, thereby directly reducing their level of meaning in life (Stillman et al., 2009). Further studies have shown that the loss of meaning resulting from social exclusion may escalate into extreme consequences such as suicidal ideation (Xue et al., 2022). Based on the above, the present study hypothesizes that social exclusion is negatively correlated with meaning in life among college students.

The mechanism by which social exclusion affects college students’ meaning in life remains to be further explored, and loneliness may serve as a mediating factor. Loneliness refers to an individual’s subjective perception of being isolated, accompanied by a sense of separation from others and negative emotional experiences arising from unmet needs for interpersonal connection (Huxhold and Fiori, 2024). According to the need-to-belong theory (Singer et al., 2022), establishing and maintaining stable, positive interpersonal relationships is a fundamental human need. When college students experience social exclusion, their need for belonging is thwarted, making it difficult for them to perceive a sense of purpose and significance from social connections (Kim and Jang, 2017; Lim et al., 2016), thereby increasing their level of loneliness (Yue and Wang, 2024). From an existential perspective, constructing and maintaining meaningful connections is a fundamental drive that enables individuals to transcend personal loneliness and affirm their own worth (Heine et al., 2006). For this reason, interpersonal relationships serve as a crucial source of meaning in life for college students. Without such positive connections, persistent loneliness may lead students into existential anxiety, causing them to feel unwanted and misunderstood, and consequently leading them to deeply question the value of their existence and the purpose of their lives, which directly reduces their sense of meaning in life (Zhang et al., 2020). In summary, the present study hypothesizes that loneliness mediates the relationship between social exclusion and meaning in life among college students.

The direct or indirect effect of social exclusion on college students’ meaning in life may also be moderated by other factors, such as nostalgia. Nostalgia is a complex emotional state triggered by recalling past experiences, events, or feelings, characterized by a bittersweet mixture of positive and negative emotions (Wildschut et al., 2010; Zhou et al., 2012). Previous studies have examined the relationships between nostalgia and social exclusion, loneliness, and meaning in life. However, the role of nostalgia as a moderator in this area remains relatively underexplored. The present study builds on this foundation to investigate the moderating mechanism of nostalgia, aiming to further clarify how nostalgia buffers or exacerbates the impact of social exclusion on individuals’ psychological adaptation. In reality, not all college students who experience exclusion develop intense loneliness; individual difference factors—particularly internal psychological resources (Guo et al., 2021)—may buffer or intensify this process. After experiencing social exclusion, individuals actively seek psychological resources to alleviate the associated distress (Williams, 2007). Nostalgia, as a longing for emotionally significant past experiences, serves as an important coping mechanism. Empirical research has found that social exclusion significantly triggers individuals’ nostalgic tendencies, because nostalgia effectively compensates for belongingness needs and provides emotional support (Zhou et al., 2008). More importantly, nostalgia is not merely a consequence of social exclusion and it also possesses significant social functions (Puente-Díaz and Cavazos-Arroyo, 2021). Through nostalgia, college students can revisit positive social connections, thereby enhancing social confidence and motivating themselves to actively seek new social bonds, which to some extent repairs the negative effects of social exclusion (Li et al., 2022). Meanwhile, as a compensatory psychological coping strategy, nostalgia contains warm interpersonal scenarios and positive self-images in its memories, effectively providing individuals with a substitute satisfaction of “social connection.” Nostalgia is not merely a passive response to loneliness; it also has proactive socio-psychological functions that can actively reduce individuals’ feelings of loneliness (Wildschut et al., 2006) and buffer against the negative emotions associated with loneliness and exclusion (Abeyta and Juhl, 2023). Therefore, nostalgia may moderate the relationship between social exclusion and loneliness in college students. Individuals with high levels of loneliness may be more prone to nostalgia (Routledge et al., 2008). When loneliness is intense, individuals with high nostalgic tendencies can actively recall past warm and meaningful experiences (e.g., joyful gatherings with family and friends, personal developmental milestones, etc.) (Newman and Sachs, 2022). These memories can instantly supplement a “virtual presence” of social connection (You and Zhong, 2022), effectively filling the belongingness gap created by current loneliness (Wildschut et al., 2010), thereby alleviating the impact of loneliness on meaning in life (Sedikides et al., 2008). In contrast, individuals with low nostalgic tendencies lack such internal psychological resources and are more likely to fall into a state of meaninglessness when experiencing loneliness (Dong et al., 2025). Consequently, nostalgia may moderate the relationship between loneliness and meaning in life among college students. For college students with high nostalgic tendencies, the negative experiences brought by social exclusion may be reappraised against the backdrop of nostalgic memories, which provide a buffering contrast. Past positive experiences offer them alternative evidence that “I am still loved and I still have value” (Sedikides and Wildschut, 2018), thereby weakening the direct damage of social exclusion to meaning in life. In contrast, low-nostalgia individuals lack such positive cognitive alternative resources in exclusionary situations, making them more likely to internalize exclusion as a negation of their own existential worth, which in turn accelerates the loss of meaning in life (Yang et al., 2023; Zhang et al., 2025). Therefore, nostalgia may also moderate the direct relationship between social exclusion and meaning in life among college students.

**Figure 1:**
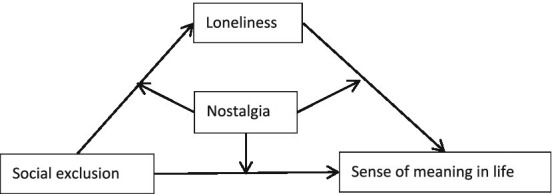
Theoretical model.

In summary, the present study proposes the following hypotheses: the direct path through which social exclusion affects college students’ meaning in life is moderated by nostalgia, and both the first and second halves of the mediating path (social exclusion → loneliness → meaning in life) are moderated by nostalgia, as illustrated in Figure 1. The core contribution of this study lies in constructing a moderated mediation model that elucidates the mechanism underlying the effect of social exclusion on college students’ meaning in life. Unlike previous research, the present study introduces nostalgia as a moderator and explores the potential “double-edged sword” effect of nostalgia within this model. This finding offers a new perspective for understanding the conditional boundaries of the impact of social exclusion on meaning in life, and also provides empirical evidence for differentiated nostalgia-based interventions in colleges and universities.

## 2 Materials and methods

### 2.1 Study design

A cross-sectional survey design was adopted in this study, with data collected via an online questionnaire. The study aimed to investigate the associations among social exclusion, loneliness, nostalgia, and meaning in life, and to evaluate the mediating effect of loneliness and the moderating effect of nostalgia. Data collection was conducted from August to October 2025.

### 2.2 Participants and inclusion criteria

College students from a university in Shanxi Province were recruited to voluntarily participate in this study. Inclusion criteria were: (1) age between 18 and 30 years, (2) current full-time college or university student, (3) owning and using a smartphone, (4) voluntarily participating in the questionnaire survey and signing the electronic informed consent form. Exclusion criteria included: (1) a history of mental disorders, (2) other addictive behaviors, (3) duplicate submissions or automated (bot) responses. The study followed the ethical principles related to work with humans as outlined in the Declaration of Helsinki.

### 2.3 Data collection procedure

An online questionnaire was administered via the mainstream Chinese survey platform “Sojump”.^1^ After clicking the link, participants first read an electronic informed consent form, which clearly explained the study content, confidentiality measures, and their right to withdraw freely. Participants were allowed to proceed to the questionnaire only after agreeing to participate and signing the electronic informed consent form.

The questionnaire incorporated several quality control measures: (1) attention check items: one or two instructional questions (e.g., “Please select ‘Agree’ for this item”) were inserted; (2) reverse-scored items: some scales included reverse-coded items; (3) response time screening: the completion time for each questionnaire was recorded, and responses with a completion time of less than 3 minutes were excluded; (4) identification of duplicate and automated responses: based on IP addresses, device IDs, and submission timestamps, duplicate submissions from the same IP address or device were excluded; additionally, pattern recognition (e.g., consecutive identical responses or obviously patterned answers) was used to identify and exclude invalid responses automatically submitted by bots.

After data collection, the raw data were exported from the online platform and stored on an encrypted hard drive, accessible only to core members of the research team. A total of 1,812 questionnaires were collected, yielding 1,714 valid responses, with an effective response rate of 94.6%.

### 2.4 Sample characteristics

Among the 1,714 participants included in the final analysis, 1,018 were male (59.40%) and 696 were female (40.6%). Participants’ ages ranged from 18 to 30 years, with a mean age of 20.77 years (SD = 2.312). The sample spanned from first-year undergraduate to third-year doctoral students.

### 2.5 Measures

#### 2.5.1 Demographic questionnaire

A self-designed demographic questionnaire was used to collect information including gender, grade, and other relevant characteristics.

#### 2.5.2 Social exclusion questionnaire for college students

The college student social exclusion questionnaire developed by Wu et al. (2013) was adopted. This 19-item scale, rated on a 5-point Likert scale, comprises two subscales: direct exclusion (10 items, items 1–10) and indirect exclusion (9 items, items 11–19). Higher scores indicate higher levels of social exclusion experienced by individuals in daily life. In the present study, the Cronbach’s *α* coefficient for this scale was 0.979, indicating excellent internal consistency. To further examine its structural validity, a confirmatory factor analysis (CFA) was conducted based on our sample data, which yielded satisfactory fit indices: CFI = 0.965, TLI = 0.961, IFI = 0.965, and RMSEA = 0.071, supporting the original factor structure of the scale.

#### 2.5.3 Loneliness scale

The Loneliness Scale revised by Wang (2016) was used in this study. This scale comprises 20 items and is rated on a 4-point Likert scale, with higher scores indicating stronger feelings of loneliness. As this scale is theoretically conceptualized as a unidimensional measure, internal consistency reliability was used as the primary psychometric indicator in the present study. The Cronbach’s alpha coefficient for this scale was 0.921, indicating good reliability. The scale has demonstrated sound psychometric properties in prior research, and the high Cronbach’s alpha in the present sample further supports its reliability.

#### 2.5.4 Meaning in life scale

The Chinese version of the Meaning in Life Scale revised by Wang et al. (1999) was employed. This 10-item scale, rated on a 7-point Likert scale, comprises two subscales: Presence of Meaning (Meaning Experience, 5 items, Items 1–5) and Search for Meaning (Meaning Seeking, 5 items, Items 6–10). Higher scores indicate higher levels of meaning in life. In the present study, the Cronbach’s *α* coefficient for this scale was 0.949, indicating excellent internal consistency. To further evaluate its structural validity, a confirmatory factor analysis (CFA) was conducted on our sample data, which yielded the following fit indices: CFI = 0.973, TLI = 0.964, IFI = 0.973, and RMSEA = 0.094. These results suggest an acceptable model fit (RMSEA < 0.10), supporting the scale’s factor structure in this sample.

#### 2.5.5 Indigenous nostalgia scale

The Localized Nostalgia Scale developed by Lu (2008) was adopted. This 14-item scale, rated on a 7-point Likert scale, comprises three subscales: Social Nostalgia (5 items, Items 5, 8, 9, 10, 12), Belief Nostalgia (4 items, Items 2, 4, 7, 11), and Object Nostalgia (5 items, Items 1, 3, 6, 13, 14). Higher scores denote higher levels of nostalgia. In the present study, the Cronbach’s *α* coefficient for this scale was 0.928, indicating good internal consistency. To further evaluate its factor structure, a confirmatory factor analysis (CFA) was conducted, which produced the following indices: CFI = 0.898, TLI = 0.875, IFI = 0.898, and RMSEA = 0.106. These values suggest that the model fit was less than optimal, which may be attributable to sample characteristics or the multidimensional nature of the nostalgia construct. Nevertheless, the scale was retained given its established theoretical basis and prior validation.

### 2.6 Data analysis

In this study, SPSS version 27.0 was used for data entry, organization, and analysis. Descriptive statistical analyses (means and standard deviations) and Pearson product–moment correlation analyses were conducted to examine the relationships among social exclusion, loneliness, nostalgia, and meaning in life. Subsequently, the PROCESS macro was employed to test the mediating effect of loneliness (Model 4) and the moderated mediating effect of nostalgia (Model 59), respectively.

## 3 Results

### 3.1 Common method Bias

Harman’s single-factor test was conducted to examine common method bias. The results revealed six factors with eigenvalues greater than 1, and the variance explained by the first common factor was 37.178%, which is below the recommended threshold of 40%. This indicates that common method bias in this study was within an acceptable range (Tang and Wen, 2020).

### 3.2 Descriptive statistics and correlation analyses of variables

As shown in Table 1, social exclusion was significantly positively correlated with loneliness, and significantly negatively correlated with nostalgia and meaning in life. Loneliness was significantly negatively correlated with nostalgia and meaning in life. Nostalgia was significantly positively correlated with meaning in life.

**Table 1:** Descriptive statistics and correlation analysis of all variables.

Variables	M ± SD	1	2	3	4
1 Social exclusion	1.873 ± 0.879	1			
2 Loneliness	1.952 ± 0.572	0.690^**^	1		
3 Nostalgia	4.894 ± 1.255	−0.174^**^	−0.220^**^	1	
4 Meaning in life	5.271 ± 1.347	−0.493^**^	−0.615^**^	0.365^**^	1

*p < 0.05, **p < 0.01, ***p < 0.001, same blow.

### 3.3 Moderated mediation effect analysis

Prior to conducting the regression analyses, the basic assumptions of linear regression were tested. Multicollinearity diagnostics revealed that the variance inflation factor (VIF) for all predictors ranged from 1.052 to 1.945, well below the threshold of 5, indicating no serious multicollinearity. Normality of residuals (Q-Q plots), linearity (residual plots), and homoscedasticity (residual plots) were visually examined and showed no significant violations, suggesting that the regression analyses satisfied the key assumptions. Correlation analysis reveals that the potential influence of gender as one of the confounding factors ranges from 0.10 to 0.30. These correlations suggest minor associations between these factors and key variables, though the effects are not considered substantial. First, Model 4 of the PROCESS macro for SPSS was used to test the mediating role of loneliness between social exclusion and meaning in life. The results are presented in Table 2. After controlling for gender and age, social exclusion significantly positively predicted loneliness (*B* = 0.449, *p* < 0.001) and significantly negatively predicted meaning in life (*B* = −0.753, *p* < 0.001). When loneliness was included as a mediator, both social exclusion (*B* = −0.206, *p* < 0.001) and loneliness (*B* = −0.516, *p* < 0.001) remained significant predictors of meaning in life. These results indicate that loneliness partially mediated the relationship between social exclusion and meaning in life, with a mediation effect value of −0.547, accounting for 72.64% of the total effect (see Table 3).

**Table 2:** Regression analysis of the mediating role of loneliness.

Outcome variable	The regression equation	Overall fitting index			Significance of regression coefficient	
	Predictors	*R*	*R^2^*	*F*	*B*	*t*
Loneliness	Gender	0.690	0.477	516.514^***^	0.049	2.436^*^
	Age				−0.003	−0.163
	Social exclusion				0.449	39.270^***^
Meaning in life	Gender	0.493	0.243	181.779^***^	0.125	−2.167^*^
	Age				0.000	−0.685
	Social exclusion				−0.753	−23.219^***^
Meaning in life	Gender	0.618	0.382	263.057^***^	−0.065	−1.239
	Age				−0.000	−0.836
	Social exclusion				−0.206	−5.102^***^
	Loneliness				−0.516	−19.599^***^

**Table 3:** Decomposition of direct and indirect effects of social exclusion on college students’ meaning in life.

Effect	Effect (*B*)	Bootstrap SE	95% bootstrap confidence intervals		Proportion of the total effect (%)
			Boot LLCI	Boot ULCI	
Direct effect	−0.206	0.040	−0.286	−0.127	27.36
Indirect effect	−0.547	0.044	−0.634	−0.462	72.64
Total effect	−0.753	0.032	−0.816	−0.689	100.00

The regression coefficients presented are unstandardized coefficients (*B*). The bootstrap method with 5,000 resamples was used to test the mediating effect. The indirect effect is considered statistically significant if the 95% bias-corrected bootstrap confidence intervals (Boot LLCI to Boot ULCI) do not contain zero.

**Figure 2:**
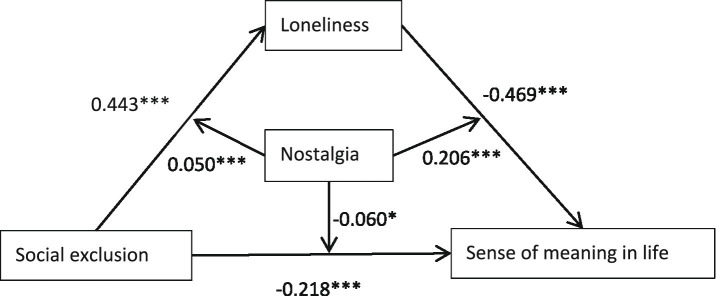
Moderated mediation model. All path coefficients shown in figure are unstandardized coefficients (B).

Subsequently, Model 59 in the PROCESS macro was used to construct a moderated mediation model, with gender and age entered as control variables, social exclusion as the independent variable, loneliness as the mediator, nostalgia as the moderator, and meaning in life as the dependent variable. The results are presented in Table 4 and Figure 2. The interaction term between social exclusion and nostalgia significantly predicted loneliness (*B* = 0.050, *p* < 0.001), indicating that nostalgia significantly moderated the relationship between social exclusion and loneliness. The interaction term between social exclusion and nostalgia also significantly predicted meaning in life (*B* = −0.060, *p* < 0.05), indicating that nostalgia significantly moderated the direct relationship between social exclusion and meaning in life. Furthermore, the interaction term between loneliness and nostalgia significantly predicted meaning in life (*B* = 0.206, *p* < 0.001), indicating that nostalgia significantly moderated the relationship between loneliness and meaning in life.

**Table 4:** Testing the moderating effect of nostalgia.

Outcome variable	The regression equation	Overall fitting index			Significance of regression coefficient	
	Predictors	*R*	*R^2^*	*F*	*B*	*t*
Loneliness	Gender	0.706	0.498	337.727^***^	0.048	2.441^*^
	Age				0.000	−0.087
	Social exclusion				0.443	38.872^***^
	Nostalgia				−0.038	−4.729^***^
	Social exclusion × nostalgia				0.050	6.157^***^
Meaning in life	Gender	0.668	0.446	195.577^***^	−0.077	−1.548
	Age				0.000	−0.427
	Social exclusion				−0.218	−5.574^***^
	Loneliness				−0.469	−18.396^***^
	Nostalgia				0.244	12.048^***^
	Social exclusion × nostalgia				−0.060	−2.463^*^
	Loneliness × nostalgia				0.206	5.500^***^

Furthermore, to clearly illustrate the simple slopes across varying levels of nostalgia, all continuous variables were Z-standardized prior to conducting the simple slope analyses. Accordingly, the simple slope effects are presented as standardized coefficients.

Simple slope analyses revealed that among college students with low levels of nostalgia (*Z* = −1), social exclusion had a significant positive effect on loneliness (*β*_simple = 0.584, *p* < 0.001) and among those with high levels of nostalgia (*Z* = 1), this effect was stronger (*β*_simple = 0.777, *p* < 0.001), and the difference was significant (*p* < 0.001). This indicates that as nostalgia increases, the effect of social exclusion on loneliness becomes stronger.

Regarding meaning in life, when nostalgia was low, social exclusion had a significant negative effect (*β*_simple = −0.093, *p* < 0.01), and when nostalgia was high, this negative effect was stronger (*β*_simple = −0.191, *p* < 0.001), and the difference was significant (*p* < 0.001). This suggests that as nostalgia increases, the negative impact of social exclusion on meaning in life becomes stronger.

Furthermore, when nostalgia was low, loneliness had a significant negative effect on meaning in life (*β*_simple = −0.579, *p* < 0.001), and when nostalgia was high, this negative effect weakened (*β*_simple = −0.359, *p* < 0.001), and the difference was significant (*p* < 0.001). This indicates that as nostalgia increases, the negative effect of loneliness on meaning in life among college students gradually weakens.

## 4 Discussion

The present study found a negative association between social exclusion and meaning in life among college students, which is consistent with previous findings (Twenge et al., 2003). From a theoretical perspective, this result aligns with self-determination theory (Petrovic et al., 2024): social exclusion is often accompanied by the frustration of individuals’ basic psychological need for interpersonal relationships, as well as a lack of belongingness and security, which are closely related to a reduced sense of meaning in life. In addition, this finding echoes the need-to-belong theory (Pardede and Kovač, 2025): humans possess a fundamental desire for stable interpersonal connections, and when such connections are difficult to obtain, individuals are more likely to experience a lower level of meaning in life. For college students, who are in a critical period of constructing self-identity, social exclusion is often accompanied by the cognition of “being rejected by others,” which is closely associated with individuals’ perceived existential value and their sense of meaning in life.

In addition, the present study identified a partial mediating effect of loneliness in the relationship between social exclusion and meaning in life among college students. Specifically, social exclusion was negatively associated with meaning in life, and this association was partially accounted for by loneliness. More precisely, social exclusion is often accompanied by profound experiences of emotional isolation and relational disconnection, and loneliness is closely related to such experiences. When individuals face these distressing psychological experiences, their original meaning system may be challenged (Heine et al., 2006). Loneliness, as a signal associated with feelings of distress, may co-occur with persistent rumination about interpersonal deficits. Such rumination tends to be accompanied by a reduction in the psychological energy required for meaning-making and the pursuit of long-term life goals, thereby relating to a decreased sense of meaning in life (Chen et al., 2023). Thus, loneliness can be regarded as an important psychological variable linking external experiences of exclusion to an internal lack of meaning in life. These findings suggest that in efforts to promote college students’ mental health and life education, we should not only strive to create an inclusive and supportive campus interpersonal environment to reduce the occurrence of social exclusion, but also pay special attention to the emotional states of those who have already experienced exclusion. Implementing effective psychological interventions, such as group counseling and mindfulness-based training (Xiang et al., 2024), to help alleviate their loneliness may contribute to repairing and enhancing their sense of meaning in life, thereby promoting their overall physical and mental health and development.

Previous research has often regarded social exclusion and loneliness as highly related concepts, or treated loneliness as a possible consequence of social exclusion (Williams, 2007). The present study found that nostalgia plays a key moderating role in distinguishing between social exclusion and loneliness—two closely related but fundamentally distinct constructs. By introducing nostalgia as a moderator, this study demonstrates the boundary conditions of the relationship between these two constructs and individuals’ psychological functioning. Specifically, under conditions of low-level social exclusion, individuals with high nostalgic tendencies reported lower levels of loneliness. This finding is consistent with the traditional positive-function view of nostalgia: as a positive emotion regulation strategy, nostalgia tends to be accompanied by the revisiting of warm past social connections, which helps compensate for minor current social threats, thereby maintaining psychological balance and being associated with lower levels of loneliness (Zhou et al., 2008). However, under conditions of high-level social exclusion, individuals with high nostalgic tendencies reported higher levels of loneliness. This finding suggests that when individuals face intense and realistic exclusion experiences, repeatedly immersing themselves in fond memories of past social relationships may create a sharp “cognitive-reality” contrast with the harsh present. According to depressive cognitive theory (Tian et al., 2023), such a contrast may activate a maladaptive ruminative thinking pattern (Zou et al., 2025)—individuals passively and repeatedly compare the past with the present. This may transform nostalgia from an adaptive emotional resource that originally had a compensatory tendency into a brooding mechanism trapped in painful comparisons (van der Velden et al., 2023), rendering it unable to exert an effective compensatory function. Instead, nostalgia may co-occur with the continued amplification of the current sense of social deficit, making individuals feel more keenly the gap and isolation of “things are not what they used to be.” Such experiences are closely associated with increased loneliness (Wildschut et al., 2006) and elevated levels of depression (Jiang et al., 2024).

In a stable environment characterized by low-level social exclusion, high nostalgia tends to be associated with individuals’ extraction of continuity and purpose from past experiences and the integration of their self-narratives, which in turn is linked to higher levels of meaning in life (Sedikides and Wildschut, 2018). Intriguingly, under the stressful condition of high-level social exclusion, high nostalgic tendencies are instead associated with lower levels of meaning in life. One possible explanation is that severe social exclusion involves the shaking of individuals’ fundamental sense of existential worth (“Am I needed?”). At this point, relying excessively on nostalgia as a way to find meaning may become ineffective due to the aforementioned “contrast effect.” When individuals find that a positive past cannot address their current existential crisis, they may experience doubts about the coherence of their self-narratives (Juhl et al., 2021), and may even perceive their past meaning as somewhat illusory. Such experiences are closely associated with an overall decline in meaning in life.

When individuals reported low levels of nostalgia, the negative association between loneliness and meaning in life was stronger; when they reported high levels of nostalgia, this negative association was significantly weaker. In other words, high nostalgia co-occurs with a weakened effect of loneliness on meaning in life, suggesting that nostalgia may serve as a protective factor that attenuates the link between loneliness and reduced meaning in life. This finding is consistent with the theoretical predictions of the Meaning Maintenance Model. According to this model, when individuals face negative states such as loneliness, their meaning system is threatened, prompting them to seek compensatory meaning resources (Heine et al., 2006). Nostalgia, as a meaning-making activity directed toward the past, is cross-sectionally associated with the retrieval of memory fragments that are purposeful, belonging-oriented, and valuable in an individual’s life, potentially providing a psychological “anchor point” for those who feel lonely (Zhang et al., 2024). Particularly among college students with high levels of loneliness, high nostalgia co-occurs with relatively higher levels of meaning in life, suggesting that nostalgia may be associated with the maintenance of meaning in life under loneliness by reinforcing self-continuity and a sense of existential worth (Yuan et al., 2025; Abeyta and Pillarisetty, 2023).

The present study deepens the understanding of coping strategies for social exclusion and loneliness. When facing social exclusion, relying solely on a single psychological priming or intervention such as nostalgia may have limitations, as its effectiveness may be moderated by the level of exclusion perceived by the individual (high vs. low). Similarly, in coping with loneliness, simply introducing nostalgia is insufficient, and the individual’s level of loneliness should be further considered. Therefore, this study advocates for a more differentiated approach in psychological intervention practice, namely implementing targeted coping strategies based on the specific levels of social exclusion and loneliness. This provides important implications for improving the precision and effectiveness of mental health work. This study has significant theoretical value and practical implications. Theoretically, by constructing a moderated mediation model, this study not only confirms that social exclusion directly weakens college students’ meaning in life, but also reveals the key mediating role of loneliness, thereby clarifying the underlying mechanism of “how it affects.” Furthermore, it innovatively identifies nostalgia as a positive psychological resource, reveals its multi-level protective effects along the above pathways, and deepens the understanding of the boundary conditions regarding “when the effect is stronger.” Practically, the findings offer clear directions for mental health work in colleges and universities: on the one hand, they suggest efforts to prevent and reduce social exclusion on campus and to pay attention to students’ loneliness; on the other hand, they provide specific and effective empirical evidence for using systematic “nostalgia-based interventions” to enhance the sense of meaning in life and strengthen the psychological resilience of college students who experience social exclusion.

## 5 Limitations and future research

First, it is also important to note that the current research did not examine the potential influence of family socioeconomic status on the four variables under investigation, which remains an open problem for future research.

Second, as the subjects of this study are college students, the sample is focused on a homogeneous group with a relatively narrow age range, so it cannot be generalized to other groups at this time. Moreover, the cross-sectional design precluded examination of temporal precedence and directional relationships among variables, thereby limiting causal inferences, and future longitudinal research is encouraged to deepen our understanding of the dynamic interplay among these constructs. Additionally, the sample was predominantly drawn from universities located in economically developed regions of China, which limits the generalizability of the findings to students from less accessible or underdeveloped areas.

Third, the effect of temporal focus on the variables examined in this study remains unverified. Future research should incorporate this critical construct to further expand the scope of the current investigation.

Forth, future research could enrich the current framework by integrating additional positive psychology constructs, such as mindfulness or self-compassion, to further enhance both the theoretical and applied value of this research domain.

Fifth, this study relied solely on “yes/no” self-report questions in an online questionnaire to exclude participants with a history of mental disorders or other addictive behaviors, which is a limitation. Self-report screening is susceptible to misclassification due to participants’ subjective interpretation bias, inaccurate recall, or tendency to conceal. Moreover, the lack of clinical confirmation of prior history and assessment of current status may compromise the purity of the sample. Future studies could consider using standardized diagnostic tools or multi-source information for exclusion to enhance internal and external validity. Additionally, all participants in this study completed the questionnaire using smartphones; therefore, we acknowledge that a small minority of students who, due to economic constraints or lifestyle choices, do not own smartphones may differ from our sample in terms of loneliness or social integration. Consequently, caution should be exercised when generalizing the present findings to college students who completely lack access to smartphones.

Sixth, the participants in this study were college students, a design that sacrifices the representativeness of the broader real-world population, particularly failing to include individuals who experience the most extreme levels of loneliness and loss of meaning in life—those most in need of understanding, such as individuals with major depressive disorder or generalized anxiety disorder. Therefore, future research should expand in at least the following two directions. First, community or clinical samples without exclusion criteria should be used to re-examine the hypothesized model and investigate whether the effect sizes are strengthened or change in form when highly symptomatic individuals are included. Second, stratified or moderated analyses should be conducted to directly compare the similarities and differences between individuals with and without a history of mental disorders along the pathway of social exclusion → loneliness → meaning-in-life crisis. Furthermore, subsequent studies may consider using more refined diagnostic interviews rather than self-reported history to more accurately delineate the boundaries and overlap between dimensions of mental illness and the core constructs.

Seventh, this study speculates four patterns: (1) Low social exclusion and low loneliness: Growth-exploration type. These clients have high external interpersonal support and subjectively do not feel lonely. The primary goal for counselors is to support personal growth. (2) Low social exclusion and high loneliness: Inner emptiness type. These clients are in a relatively friendly external interpersonal environment but are dissatisfied with existing relationships and lack deep connections. Counselors can guide them to recall warm moments of unconditional acceptance and love, exploring “What characterized your past positive relationships?” to deepen self-other understanding and alleviate loneliness. (3) High social exclusion and high loneliness: Crisis intervention type. These clients often experience a stressful external interpersonal environment and are overwhelmed by inner loneliness and distress. Counselors should temporarily avoid using nostalgia directly, prioritize crisis intervention and emotional stabilization, and after the individual’s emotions have calmed, they can guide the individual to recall “How have I overcome similar difficulties in the past?” to restore confidence and gradually recover mental health. (4) High social exclusion and low loneliness: Psychological resilience type. These clients are objectively in a rejected or isolated environment (e.g., changing majors, studying away from home), but subjectively do not feel lonely. Their support system may not be physically present (e.g., distant family members, friends elsewhere), yet they are internally strong and fulfilled, possessing a rich inner world, firm beliefs, or independent spiritual pursuits. Counselors can help them recall figures or stories that have given them strength in life, drawing spiritual support from those memories. However, these are only the researchers’ speculations and not empirical findings of the present study, and they remain a direction for future research. For example, based on the present study, qualitative research could be added to further advance the field.

Eighth, the age range of the sample in this study spanned 18 to 30 years, covering first-year undergraduates to doctoral students. Although we statistically controlled for age as a continuous variable in the regression models, controlling only for the linear effect of age may not be sufficient to eliminate fundamental differences in psychosocial environment, stressors, and sources of meaning in life among groups at different educational stages. Future research should further examine the potential moderating role of developmental stage in the present model through educational-level subgroup analyses or nonlinear modeling.

Finally, regarding the measurement instruments, the Nostalgia Scale developed by Lu (2008) and adopted in this study was constructed in the early 2000s, more than 15 years ago. The item contexts (e.g., “indifference of human feelings,” “pace of life,” and “carefree times”) are primarily rooted in the Chinese social consumption context and interpersonal interaction patterns of that period. However, contemporary college students have grown up in a digital era characterised by highly developed mobile internet and social media. With the shift from “traditional neighbourhood communities” to “digital simulated environments,” their cognitive schemas concerning “interpersonal boundaries” and “perceptions of social change” have undergone substantial shifts. The nostalgic triggers in traditional real-world communities are qualitatively different from the emotional bonding patterns in current digital simulated environments, which poses potential challenges to the semantic validity of the original items. The confirmatory factor analysis (CFA) results for this scale in the present study showed CFI = 0.898, TLI = 0.875, IFI = 0.898, and RMSEA = 0.106. Although the CFI value approached the acceptable threshold of 0.90, and the Cronbach’s *α* coefficient for this scale was 0.928 in this study, indicating excellent internal consistency (i.e., good homogeneity) among items, the relatively high RMSEA and the TLI slightly below the ideal cutoff suggest that the model’s exact fit was less than satisfactory. Notably, this discrepancy may be partially attributable to the relatively large sample size of this study (*N* = 1,714). In structural equation modelling, absolute fit indices such as RMSEA are highly sensitive to the discrepancy between the model-implied covariance matrix and the saturated matrix; with a large sample, the chi-square value tends to inflate, which in turn systematically overestimates RMSEA—a common statistical phenomenon in large-sample studies. Nevertheless, considering all the indices together, the construct validity of this classic scale still exhibited a certain degree of attenuation among contemporary college students. Meanwhile, building upon the present findings, future researchers are advised to combine cognitive interviewing and differential item functioning (DIF) analysis. After updating the item contexts to incorporate elements of digital social interaction and simulated environments, subsequent studies should re-examine the reliability and validity of the scale across different age groups and cohorts, so as to systematically evaluate the measurement equivalence of this construct in the new era, thereby providing a more robust measurement tool for nostalgia research.

## Data Availability

The data presented in this study are available on request from the corresponding author. The data are not publicly available due to ethical restrictions and participant privacy.

## Footnotes

1
https://www.wjx.cn
